# Erdheim-Chester manifesting purely as a neurological disease

**DOI:** 10.1055/s-0045-1809403

**Published:** 2025-06-21

**Authors:** Lídia Laura Salvador Ramos, Iago Resende Carvalho, Diogo Fernandes dos Santos, Andrea de Martino Luppi, Bruno de Carvalho Dornelas

**Affiliations:** 1 Universidade Federal de Uberlândia, Hospital de Clínicas, Departamento de Diagnóstico por Imagem, Uberlândia MG, Brazil.; 2 Universidade Federal de Uberlândia, Faculdade de Medicina, Uberlândia MG, Brazil.; 3 Universidade Federal de Uberlândia, Hospital de Clínicas, Departamento de Neurologia, Uberlândia MG, Brazil.; 4 Universidade Federal de Uberlândia, Hospital de Clínicas, Departamento de Anatomia Patológica, Uberlândia MG, Brazil.

**Figure 1:**
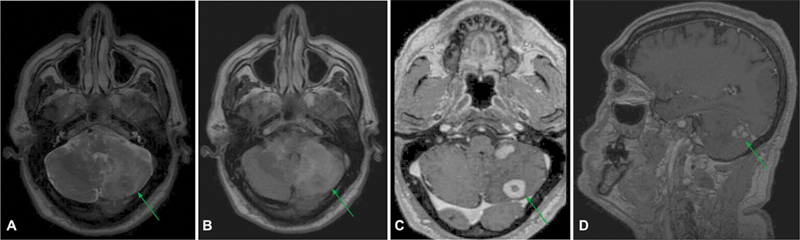
Magnetic resonance imaging scan showing expansive intraparenchymal nodular lesions located in the posterior fossa, involving the pons, peduncles, and cerebellar hemispheres, presenting mild hypointense signal in the T2-weighted sequence ( **A** ) and vasogenic edema characterized by marginal fluid-attenuated inversion recovery (FLAIR) hypersignal ( **B** ) and intense contrast enhancement ( **C,D** ).

**Figure 2:**
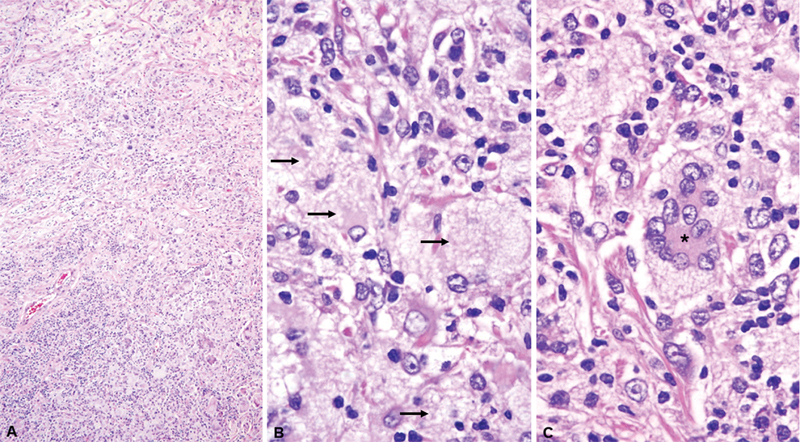
Erdheim-Chester disease in the cerebellum. ( **A** ) An infiltrate of histiocytes with a bland appearance and giant cells associated with a sparse lymphoplasmacytic infiltrate (hematoxylin and eosin staining; magnification: 4x). ( **B** ) Histiocytes characterized by abundant foamy (xanthomatous) (arrows) cytoplasm with surrounding fibrosis (hematoxylin and eosin staining; magnification: 100x). ( **C** ) Touton giant cells are frequently present(*) (hematoxylin and eosin staining; magnification: 100x).

**Figure 3:**
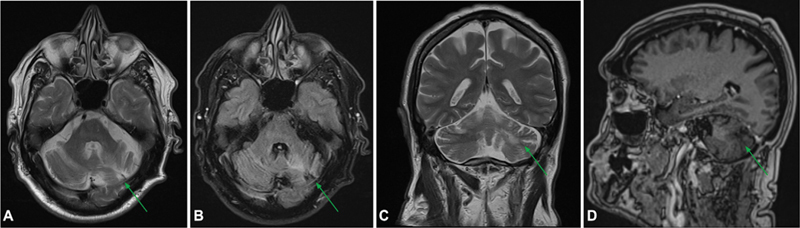
Images after the treatment with vemurafenib showing an area of sequelae in the left cerebellar hemisphere, characterized by volumetric reduction and T2/FLAIR hyperintensity ( **A–C** ), with resolution of expansile lesions in the posterior fossa and regression of enhancement ( **D** ).

A 60-year-old man presented with vertigo, paraparesis, and dysarthria that evolved over 3 months. A magnetic resonance imaging (MRI) scan revealed nodular masses in the pons, peduncles, and cerebellar hemispheres, indicating a metastatic neoplasm ( Figure 1 ). A craniectomy was performed, showing dense histiocyte proliferation, Touton giant cells, and lymphocytic infiltrate consistent with Erdheim-Chester disease ( Figure 2 ). The cytogenetic analysis confirmed a BRAF V600 mutation. Vemurafenib therapy was initiated. Six months later, the patient still exhibited dyslalia and left hemiparesis, and a follow-up MRI scan was performed, revealing an area of sequelae ( Figure 3 ). Erdheim-Chester disease is a rare non-Langerhans histiocytosis, with neurological symptoms occurring in 41% of the cases. 1 2
